# Reelin Secreted by GABAergic Neurons Regulates Glutamate Receptor Homeostasis

**DOI:** 10.1371/journal.pone.0005505

**Published:** 2009-05-11

**Authors:** Cecilia Gonzalez Campo, Mélanie Sinagra, Danièle Verrier, Olivier J. Manzoni, Pascale Chavis

**Affiliations:** 1 INSERM U862, Neurocentre Magendie, Pathophysiology of Synaptic Plasticity, Bordeaux, France; 2 Université de Bordeaux, Bordeaux, France; Tokyo Medical and Dental University, Japan

## Abstract

**Background:**

Reelin is a large secreted protein of the extracellular matrix that has been proposed to participate to the etiology of schizophrenia. During development, reelin is crucial for the correct cytoarchitecture of laminated brain structures and is produced by a subset of neurons named Cajal-Retzius. After birth, most of these cells degenerate and reelin expression persists in postnatal and adult brain. The phenotype of neurons that bind secreted reelin and whether the continuous secretion of reelin is required for physiological functions at postnatal stages remain unknown.

**Methodology/Principal Findings:**

Combining immunocytochemical and pharmacological approaches, we first report that two distinct patterns of reelin expression are present in cultured hippocampal neurons. We show that in hippocampal cultures, reelin is secreted by GABAergic neurons displaying an intense reelin immunoreactivity (IR). We demonstrate that secreted reelin binds to receptors of the lipoprotein family on neurons with a punctate reelin IR. Secondly, using calcium imaging techniques, we examined the physiological consequences of reelin secretion blockade. Blocking protein secretion rapidly and reversibly changes the subunit composition of N-methyl-D-aspartate glutamate receptors (NMDARs) to a predominance of NR2B-containing NMDARs. Addition of recombinant or endogenously secreted reelin rescues the effects of protein secretion blockade and reverts the fraction of NR2B-containing NMDARs to control levels. Therefore, the continuous secretion of reelin is necessary to control the subunit composition of NMDARs in hippocampal neurons.

**Conclusions/Significance:**

Our data show that the heterogeneity of reelin immunoreactivity correlates with distinct functional populations: neurons synthesizing and secreting reelin and/or neurons binding reelin. Furthermore, we show that continuous reelin secretion is a strict requirement to maintain the composition of NMDARs. We propose that reelin is a trans-neuronal messenger secreted by GABAergic neurons that regulates NMDARs homeostasis in postnatal hippocampus. Defects in reelin secretion could play a major role in the development of neuropsychiatric disorders, particularly those associated with deregulation of NMDARs such as schizophrenia.

## Introduction

Reelin is a large secreted protein of the extracellular matrix [1] and a signaling molecule whose best characterized pathway involves two lipoprotein receptors, apolipoprotein E receptors 2 (ApoER2) and very-low-density lipoprotein receptor (VLDLR) [2], [3]. In addition to ApoER2/VLDLR, reelin binds the cell adhesion molecule of the integrin family, α3β1 [4]. Understanding the roles of reelin, is one important key to deciphering the functions of the extracellular matrix, a molecular ensemble that forms an intricate mesh around neurons.

In the central nervous system, the spatiotemporal expression of reelin varies markedly from the prenatal to adult stage. Although the vast majority of Cajal-Retzius cells which express reelin during embryonic development [5], [6] die after the first postnatal week [7], reelin expression persists in postnatal and adult rodent brain [5], [8], [9]. At these later stages, reelin protein and mRNA localize to a subset of GABAergic neurons in the hippocampus [5], [9], [10]. Reelin secretion is independent of Ca^2+^, ionotropic glutamate receptors activity [11] and of neuronal activity [12]. Despite the proposed constitutive mode of reelin secretion [11], the characterization of neurons secreting and/or binding reelin in the postnatal brain remains to be definitively addressed. Moreover, the physiological outcome of the continuous secretion of reelin in the postnatal brain is unknown.

Reelin serves different functions during brain development and adulthood. During embryonic development, reelin is crucial to the correct cytoarchitecture of laminated structures [13], [14]. At postnatal and adult stages, reelin modulates synaptic plasticity [15], [16] and dendritic growth [17]. We have recently shown that reelin governs the maturation and surface mobility of glutamate receptors of the N-methyl D-aspartate (NMDA) subtype in the hippocampus [12], [18]. Thus, understanding the functional significance of the complex expression pattern of reelin in postnatal neurons will aid in unraveling the roles of this extracellular matrix protein in the adult brain.

Here, we demonstrate that in hippocampal cultures, reelin is synthesized and secreted by GABAergic neurons. We show that continuous reelin secretion is necessary to maintain the subunit composition of NMDARs. These data are consistent with the hypothesis that reelin is a transneuronal messenger that regulates glutamate receptor homeostasis in post-natal hippocampal neurons.

## Results

### Different types of reelin immunoreactivity coexist in the hippocampus in vitro

**Figure 1:**
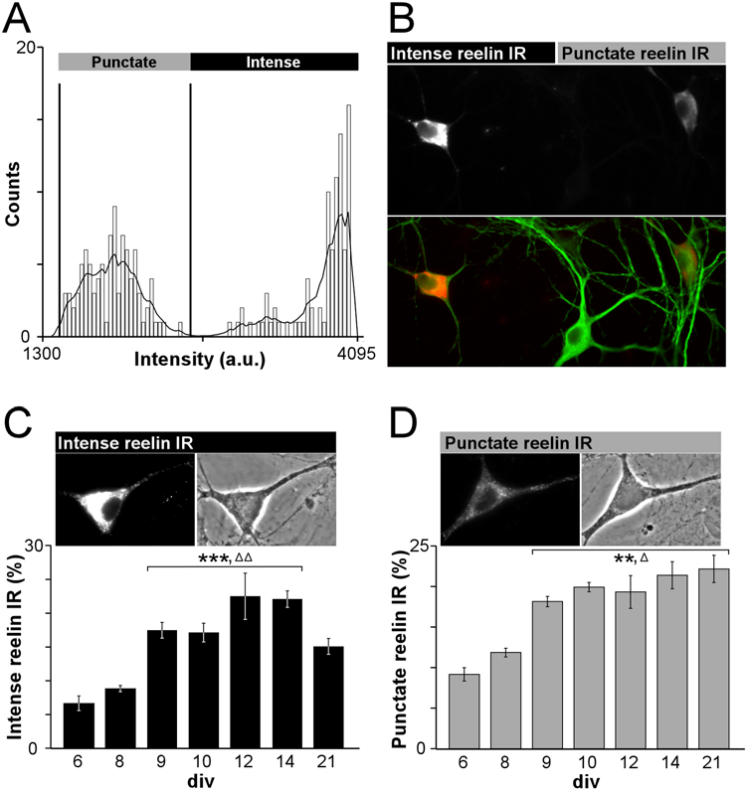
Profile of intense and punctate reelin IR during hippocampal maturation. (A) Bins of average fluorescent intensities detected in somata of 12 div hippocampal neurons immunostained for reelin (n = 380 neurons). Intensities ranging from 100 to 4030 were divided into 110 bins and the number of somata was counted within each bin. The vertical lines mark the threshold values used to identify the different reelin-positive populations (1450 and 2650). (B) Top: Representative image of somatic reelin immunoreactivities found in 12 div hippocampal neurons. Bottom: reelin immunofluorescence (red) overlaid with MAP2 counterstain (green). Neurons with intense and punctate reelin IR are present in the same field as neurons with no detectable reelin IR. (C, D) Expression profiles between 6 and 21 div, of intense (C, P<0.001: ***versus 6 and 8 div, ΔΔ versus 21 div) and punctate reelin IR (D, P<0.01: **versus 6 div, Δ versus 8 div). Bar graphs represent the average percentage±sem of reelin-positive neurons within the total number of neuronal somata counted on the corresponding phase-contrast images. High magnification images of 12 div neurons displaying intense (C) or punctate reelin IR (D) are shown on the top. Images were taken from the same coverslip with identical exposure times.

We analyzed, during maturation, the patterns of reelin expression in cultured hippocampal neurons with the G10 antibody [9], [18]. Two types of immunoreactivities and expression profiles were consistently observed in neuronal somata between 6 and 21 days in vitro (div, Figure 1). The distribution of staining intensities discriminated two distinct groups of neurons (Figure 1A). One population was characterized by its diffuse and intense perinuclear immunoreactivity (IR) and was referred to as neurons with “intense reelin IR” (Figure 1B, C). In contrast, the second population displayed a punctate and less intense labelling that was named “punctate reelin IR” (Figure 1B, D). Regardless of intense or punctate reelin IR, some somata showed punctate labelling extending into neurites (Figure 1C, D).

The expression profiles of these two types of IR varied similarly between 6 and 14 div (Figure 1C, D). The proportion of neurons with intense reelin IR significantly increased between 8 and 9 div (8.9±0.5%, n = 10 versus 17.5±1.2%, n = 11) and stabilized between 9 and 14 div (22.1±1.2%, n = 17, F_(4,44)_ = 16.7, P<0.001; Figure 1C; [18]). The expression profile of the population with punctate reelin IR followed the same pattern between 6 and 14 div (9.2±0.8%, n = 9 versus 21.4±1.7%, n = 17; Figure 1D). At 21 div, the percentage of neurons with punctate reelin IR remained stable with regard to 9–14 div (22.2±1.7%, n = 17) whereas it decreased for intense reelin IR (15.1±1.2%, n = 17; Figure 1C, D). Thus, two distinct types of reelin immunostaining are present throughout hippocampal maturation.

Although, these two types of labelling have been reported in vivo in different brain areas including the hippocampus of adult rodents [9], their physiological roles remain unknown. We next examined the physiological role of reelin-expressing neurons at the peak of reelin expression between 9 and 15 div.

### Neurons secreting reelin are GABAergic

**Figure 2:**
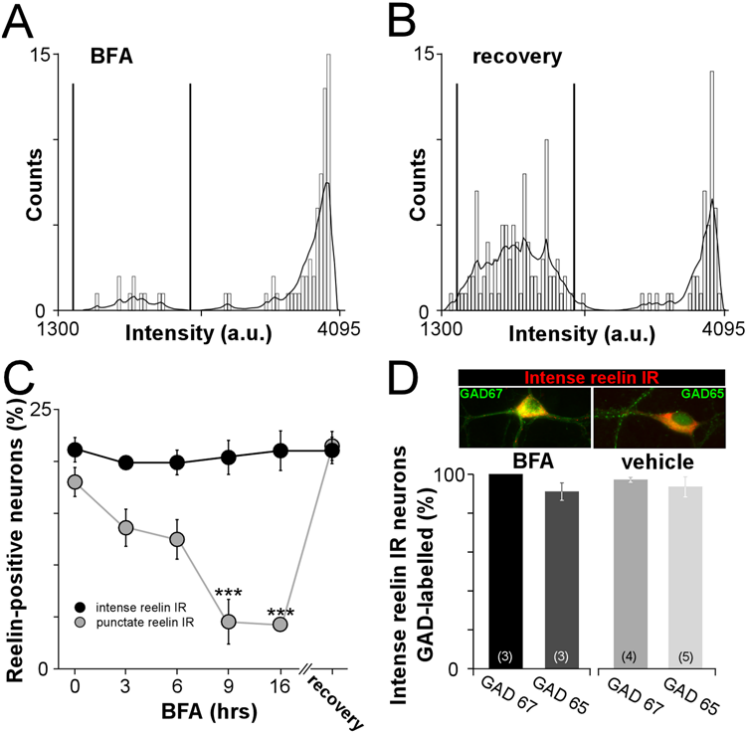
Reelin is secreted by GABAergic neurons. (A, B) Distribution of average intensities measured on neuronal somata immunolabelled for reelin after 9 and 16 hours of BFA treatment (n = 231 neurons, A) and after wash-out of BFA (n = 282 neurons, B). The intensity distribution was altered after BFA treatment (A), and the loss of the low intensity group was reverted following BFA washout (recovery, B). (C) Time course of the effect of BFA treatment on reelin IR populations. Treatment with BFA did not change the proportion of intense reelin IR neurons: 21.1±1.2% before treatment (n = 8) and 21.0±1.9% after 16 hours (n = 6), whereas it caused a significant decrease in the fraction of punctate reelin IR neurons. Note the recovery of the fraction of punctate reelin IR neurons after BFA wash-out. Values are mean percentage±sem of the total number of neuronal somata. ***P<0.001. (D) Top: Representative images of intense reelin IR (red) with GAD67 (green, left) or GAD65 (green, right). Bottom: Average percentage±sem of intense reelin IR neurons colabelled with either GAD67 or GAD65 after BFA or vehicle treatment. Values were 100.0±0.0% and 91.1±4.5% (n = 3 and 3) in BFA-treated conditions, 97.2±1.3% and 93.6±5.2% (n = 4 and 5) in vehicle conditions.

To discriminate between the intracellular and secreted form of reelin, protein secretion was blocked with brefeldin A (BFA), an inhibitor of protein translocation from the endoplasmic reticulum to the Golgi apparatus [19]. BFA dramatically altered the distribution of reelin staining intensities (Figure 2A) but not the density of reelin immunoreactivity (Figure S1A) and specifically abolished the detection of punctate reelin IR (Figure 2A, C). The time course of action of BFA shows that the fraction of punctate reelin IR neurons after 9 and 16 hours of BFA treatment was decreased to 4.5±2.1% and 4.3±0.5% (n = 5 and 6), values significantly different from 18.0±1.4% before treatment (n = 8; F_(2,16)_ = 33.05, P<0.001). The BFA-dependent blockade was fully reversible following drug washout: the fraction of neurons with punctate reelin IR following BFA recovery was not different from the value obtained prior treatment (21.5±1.4%, n = 6 versus 18.0±1.4%, n = 8; Figure 2B, C). The vehicle had no effect on either population (Figure S1B). To confirm that reelin secretion was blocked by BFA, the levels of secreted reelin were measured in the culture medium by western blot, at different times following BFA treatment (Figure S2 and Text S1). The content of secreted reelin was dramatically decreased after 3 and 9 hours of BFA treatment compared to vehicle (Figure S2B, C). Altogether, these results show that secretion blockade selectively abolished punctate reelin IR without altering the intense reelin IR population. These data suggest that intense reelin IR neurons specifically secrete reelin.

We next examined whether neurons secreting reelin were associated with a selective neuronal subtype. Double labeling of reelin with GAD67 or GAD65, two different isoforms of glutamate decarboxylase, showed that virtually all intense reelin IR were GAD67- or GAD65-positive (Figure 2D and Table 1). To determine whether intense reelin IR could be present in excitatory neurons, we performed double immunostaining of reelin with the vesicular glutamate transporters, VGlut1 or VGlut2. The immunoreactivity for VGlut1 and VGlut2 was predominantly distributed along neurites. Therefore, in most cases determining whether VGlut1- or VGlut2-positive neurites belong to intense reelin IR somata was very ambiguous. However, the few intense reelin IR somata from which neurites could be tracked with sufficient confidence (n = 12) were mostly VGlut negative (n = 9). A small population (n = 3) displayed a faint VGlut staining that was just above background threshold. To further prove that neurons secreting reelin are exclusively GABAergic, dual labeling of reelin with GAD67 or GAD65 was performed in conditions in which only intense reelin IR neurons are present. To obtain a homogeneous population of intense reelin IR neurons, we treated cultures with BFA. In these conditions, neurons with intense reelin IR were strictly co-labelled with either GAD67 or GAD65 (Figure 2D). Altogether, these data show that intense reelin IR is associated to GABAergic neurons and indicate that reelin is secreted by GABAergic neurons in postnatal hippocampus. In accord with our observations, reelin expression has previously been reported to colocalize with GABAergic markers in the hippocampus of adult rodents [5], [9], [10].

**Table 1:** Hippocampal neurons displaying intense reelin IR are GABAergic.

div	Intense reelin IR neurons (%) coexpressing		n	
	GAD 67	GAD 65	GAD 67	GAD 65
6	93.9±3.2	96.3±3.7	3	3
8	97.2±2.8	97.2±2.8	3	4
9	98.6±1.4	96.8±1.9	4	4
14	95.1±2.8	93.6±5.2	9	5
21	96.0±2.2	74.6±12.3	10	3

Average percentage±sem of intense reelin IR neurons colabelled either with GAD67 or GAD65 during in vitro maturation in untreated conditions.

### Reelin immunoreactivity segregates two distinct functional populations

**Figure 3:**
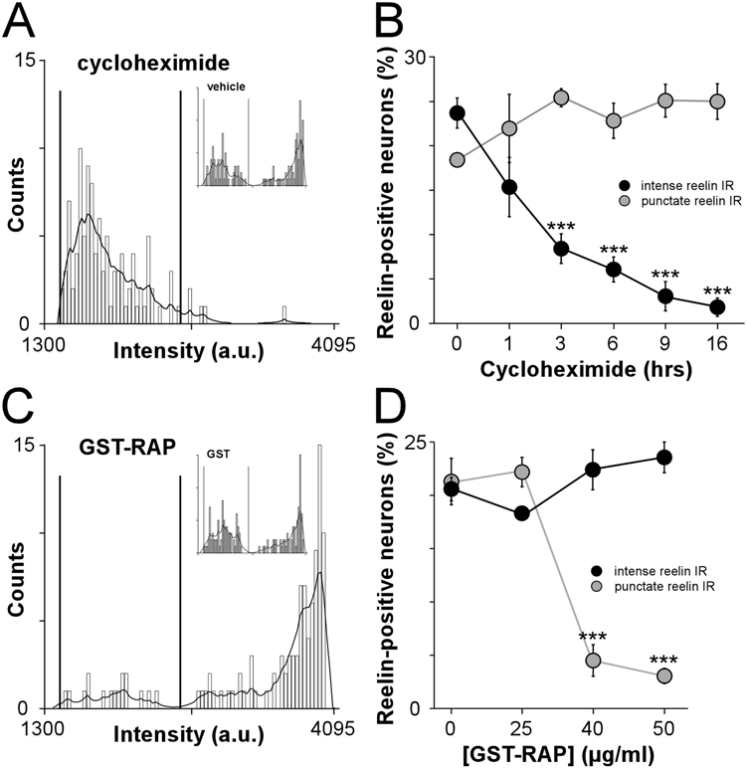
Intense reelin IR neurons synthesize reelin whereas punctate reelin IR neurons are characterized by the presence of receptor-bound reelin. (A) Distribution of average intensities of reelin IR measured on somata of neurons treated with cycloheximide (n = 270 neurons) or vehicle (inset, n = 240 neurons) for 9 and 16 hours. Cycloheximide altered the distribution of average intensities corresponding to intense reelin IR whereas it had no effect on the distribution of lower intensities. Inset: the vehicle did not affect the distribution of reelin IR intensities. (B) Time course of the effect of cycloheximide on the proportion of intense and punctate reelin IR. Cycloheximide had no effect on the punctate reelin IR population whereas it progressively decreased the fraction of intense reelin IR neurons. ***P<0.001. (C) Distribution of average intensities of reelin immunofluorescence measured on neuronal somata after treatment with 40 or 50 µg/ml of GST-RAP (n = 393 neurons) or GST alone at the same concentration (inset, n = 382 neurons). Treatment with GST-RAP altered the distribution of low intensities whereas GST had no effect. (D) Dose dependent effect of GST-RAP on the punctate reelin IR population. GST-RAP was used at 25, 40 and 50 µg/ml. A significant decrease of punctate reelin IR was observed with 40 and 50 µg/ml of GST-RAP (4.5±1.5% and 3.1±0.7%; n = 3 and 3) whereas no effect was detected with a concentration of 25 µg/ml compared to untreated (22.2±1.4% and 21.3±2.2%; n = 3 and 3; F_(3,8)_ = 45.7, ***P<0.001). Increasing concentrations of GST-RAP had no effect on the fraction of intense reelin IR neurons. Values are mean percentage±sem of the total number of neuronal somata.

To determine whether intense reelin IR neurons synthesize reelin, we studied the time course of action of cycloheximide, an inhibitor of protein synthesis [20]. Inhibition of protein synthesis progressively abolished intense reelin IR without affecting the punctate reelin IR (Figure 3A, B and Figure S3A). Intense reelin IR was significantly decreased within 3 hours of treatment (8.4±1.7%, n = 3) compared to untreated (23.6±1.7%, n = 4) or 1 hour (15.3±3.3%, n = 3; F_(2,7)_ = 12.0, P<0.01) and was abolished after 9 and 16 hours (3.1±1.6% and 1.8±1.0%, n = 3 and 3, P<0.001). The vehicle had no effect on either reelin-positive populations (Figure 3A inset and Figure S3B, C). We concluded that the intense reelin IR pattern is associated with synthesis functions.

Since neurons with punctate reelin IR are BFA-sensitive, we next challenged the hypothesis that the punctate reelin IR was associated with receptor-bound reelin. Secreted reelin binds ApoER2 and VLDLR which are widely expressed by hippocampal neurons in culture [18]. BFA is a broad spectrum inhibitor of secretion and a potential pitfall stemming from BFA experiments (Figure 2) is that blocking secretion could have reduced the number of neurons expressing ApoER2 and/or VLDLR thereby causing a decrease in punctate reelin IR. This possibility was ruled out because BFA treatment did not affect either ApoER2 or VLDLR labeling (ApoER2-positive neurons: untreated 68.9±2.1%, vehicle 68.1±2.1%, BFA 69.1±4.4%, n = 3; VLDLR-positive neurons: untreated 24.7±1.8%, vehicle 24.9±1.6%, BFA 30.5±8.4%, n = 3). To examine whether the punctate reelin labeling was due to reelin bound to receptors, hippocampal neurons were treated with the Receptor Associated Protein fused with GST (GST-RAP). RAP is a molecular chaperone for the low-density lipoprotein receptor family and is widely used as a functional lipoprotein antagonist [21]. It prevents the binding of reelin to lipoprotein receptors [22], in turn disrupting the physiological functions of reelin [12], [17], [18], [23]. GST-RAP selectively abolished punctate reelin IR in a dose dependent manner but had no effect on the intense reelin IR population (Figure 3C, D). The control GST was devoid of effect on both types of reelin-immunoreactive neurons (Figure 3C inset). Thus, preventing the binding of reelin to its lipoprotein receptors mimicked the effect of secretion blockade. In contrast, blocking reelin receptors of the integrin family did not affect reelin IR (Table S1) [24]. These results demonstrate that the punctate reelin IR population is formed by neurons that bind secreted reelin onto lipoprotein receptors.

Altogether, these data show that intense and punctate reelin IR discriminate two distinct neuronal populations: reelin is synthesized and secreted by intense reelin IR neurons that are GABAergic, whereas punctate reelin IR neurons are characterized by the presence of receptor-bound reelin.

### Characterization of punctate reelin IR neurons

To examine the neuronal subtype associated to the punctate reelin IR population, we performed colabellings of reelin with GAD65 (Table 2) or VLDLR (Figure S4). First, in contrast to the intense reelin IR population, the fraction of punctate reelin IR neurons expressing GAD65 represented an average of 54% between 6 and 21 div (Table 2). In addition, following 9 hours treatment with cycloheximide, a condition in which all reelin-positive neurons are punctate, a similar distribution was observed (i.e. 50.2±5.1% of punctate reelin IR neurons were GAD65 immunoreactive, n = 3). Thus, punctate reelin IR neurons are equally distributed between GABAergic and non-GABAergic populations. Second, dual labeling of reelin with VLDLR showed that virtually all punctate reelin IR neurons co-express VLDLR (100.0±0.0%, n = 3; Figure S4). These data suggest that regardless of their GABAergic or non GABAergic phenotype, punctate reelin IR neurons which result from receptor-bound reelin, express the reelin receptor VLDLR.

**Table 2:** Fraction of punctate reelin IR hippocampal neurons expressing GAD65.

div	Punctate reelin IR neurons expressing GAD65 (%)	n
6	41.7±8.3	3
8	52.0±14.9	4
9	56.2±9.5	4
14	59.1±4.6	5
21	61.7±15.5	3

Average percentage±sem of punctate reelin IR neurons colabelled with GAD65 during in vitro maturation in untreated conditions.

### Secretion blockade reversibly changes the subunit composition of functional NMDARs

We next searched for the physiological function of the continuous secretion of reelin by a subpopulation of hippocampal GABAergic neurons. NMDA receptors (NMDARs), a subclass of the ionotropic excitatory L-glutamate receptors, are heteromeric channels of obligatory NR1 subunits in combination with either NR2 (NR2A-NR2D) or NR3 subunits [25]. In the mammalian forebrain, NMDAR assembly is regulated during development from predominance of NR2B-containing to NR2A-containing NMDARs [18], [24], [26]–[28]. This process is governed by activity, experience [24], [29]–[31] and also by the extracellular environment, including cell adhesion molecules and the extracellular matrix protein reelin [12], [18], [24]. Here, we examined the functional consequences of reelin secretion blockade on the subunit composition of NMDARs.

**Figure 4:**
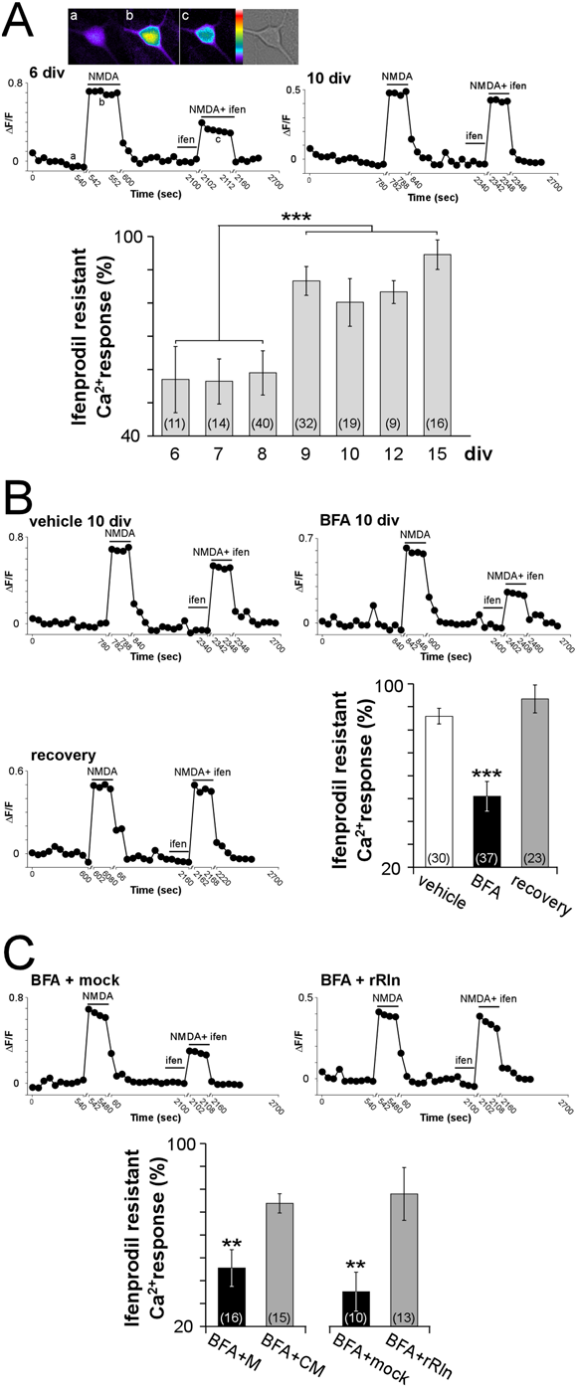
The increase of NR2B-mediated NMDARs Ca^2+^ responses induced by BFA is rescued by reelin. (A) Examples of Ca^2+^ responses recorded in a 6 div (top) or 10 div neuron (bottom). At 6 div, the NMDA-induced Ca^2+^ influx in the presence of ifenprodil (3 µM) was largely reduced compared to the response evoked by NMDA alone (100 µM). Inset: pseudocolor scale with images of Ca^2+^ responses taken at the time points indicated below. At 10 div the effect of ifenprodil on the NMDA-evoked response was less prominent. Bar graph representing average percentage of NMDA-induced Ca^2+^ influx remaining after ifenprodil application during maturation. Values were 57.0±10.0% at 6 div, 56.5±6.8% at 7 div, and 59.0±6.6% of control response at 8 div. They were significantly different between 9 and 15 div: 86.6±4.2% at 9 div, 80.2±7.2% at 10 div, 83.3±3.4% at 12 div and 95.3±4.7% at 15 div (F_(6,130)_ = 17.2, ***P<0.001). (B) Examples of NMDA-stimulated Ca^2+^ responses recorded in neurons either treated with vehicle or BFA for 15 hours or after recovery from BFA blockade. In BFA-treated conditions, the NMDA-induced Ca^2+^ influx in the presence of ifenprodil (3 µM) was largely decreased compared to the response evoked by NMDA alone (100 µM). After recovery, the effect of ifenprodil was dramatically reduced compared to BFA-treated conditions. Bar graph summarizing the average percentage of NMDA-evoked Ca^2+^ responses remaining after ifenprodil application in the conditions described above (vehicle: 86.0±3.3%, BFA: 51.0±6.5% and recovery: 93.4±6.1% of control response, F_(2,87)_ = 40.8, ***P<0.001). (C) Examples of NMDA-stimulated Ca^2+^ responses recorded after incubation of BFA-treated neurons with rRln (BFA+rRln) or the control mock (BFA+mock). In the presence of BFA+rRln, the effect of ifenprodil was largely reduced compared to BFA+mock conditions. Bar graph summarizing rescue experiments using conditioned medium (BFA+CM) or recombinant reelin (rRln). Respective controls for conditioned medium and rRln were medium not exposed to cultured neurons (M) or mock (mock). Experiments were performed at 9, 10 and 15 div. **P<0.01. The number of neurons recorded in each condition is indicated.

NMDAR channels display a high permeability to calcium (Ca^2+^). NMDARs activation was monitored by the increase of intracellular Ca^2+^ concentration upon NMDA application using the fluorescent Ca^2+^ indicator, Oregon Green BAPTA1-AM. The sensitivity of NMDA-stimulated Ca^2+^ influx to the antagonist ifenprodil was used to quantify the fraction of NR2B-containing NMDARs (Figure 4). Ifenprodil at 3 µM, provides a maximal and selective block of NR1/NR2B diheteromeric receptors [32]. As expected from the developmental loss of NR2B subunits [18], [30], [33], [34], the sensitivity of the NMDA-stimulated Ca^2+^ influx to ifenprodil was substantially greater in 6 div neurons compared to 10 div neurons (Figure 4A). The effect of ifenprodil was not the result of dye bleaching since two sequential NMDA stimulations induced identical Ca^2+^ elevations (Figure S5).

We examined whether blocking secretion with BFA affected the subunit composition of 10 div-NMDARs. In 10 div neurons treated with BFA for 15 hours, the NMDA-evoked Ca^2+^ responses were much more sensitive to the NR2B antagonist compared to vehicle-treated or untreated neurons (Figure 4B). Remarkably, the effect of BFA was fully reversible within 24 hours of BFA wash-out (Figure 4B). Altogether, these data show that secretion blockade increased the fraction of functional NR2B-containing NMDARs. We next examined whether continuous reelin secretion is necessary to maintain lower fractions of functional NR2B-containing NMDARs.

### Reelin secretion is necessary to maintain the subunit composition of NMDARs

Brefeldin A is a non-selective inhibitor of protein secretion. To demonstrate that the lack of reelin secretion caused the increase in the fraction of NR2B-containing NMDARs, we performed rescue experiments. We examined whether endogenously secreted reelin or exogenous recombinant reelin was sufficient to rescue the BFA-dependent effects. Neurons were treated with BFA alone during 9 hours; then incubated, in the continuous presence of BFA, with reelin for the following 13 hours. Endogenous reelin secreted by hippocampal neurons was present in conditioned medium collected from 16–17 div cultures (BFA+CM, see materials and methods), and exogenous recombinant reelin was added at a concentration of 2.50–5.55 nM (BFA+rRln; [18]). In these conditions, the NMDA-evoked Ca^2+^ responses were much less sensitive to ifenprodil compared to their respective control (73.8±4.2% in BFA+CM versus 45.5±8.0% in BFA+M; 78.0±11.5% in BFA+rRln versus 35.2±8.5 in BFA+mock, Figure 4C). These data demonstrate that soluble reelin is necessary and sufficient to rescue the effects of secretion blockade and to regulate NMDARs homeostasis.

## Discussion

Our study provides new insights into the interactions between extracellular matrix proteins and glutamate receptors. First, we demonstrate that two distinct neuronal populations of expressing reelin IR coexist in the hippocampus in vitro to serve different functions. A subpopulation of purely GABAergic neurons synthesize and secrete reelin which in turn binds the lipoprotein receptors of another neuronal subpopulation. Second, we discovered that continuous reelin secretion is a strict requirement to maintain the composition of NMDARs.

The two types of somatic reelin IR we described here have been reported in different brain areas of adult primates [35]. Whether they also correlate with distinct physiological functions in the primate brain is unknown. During embryonic development, reelin is secreted by Cajal-Retzius neurons [1], [13]. Recent evidences indicate that Cajal-Retzius neurons are glutamatergic [36], [37]. Together with our results, these data suggest that the multiple and different functions of reelin during development and adulthood are linked to diverse reelin producing cell types. Further studies will be necessary to determine whether GABAergic neurons also produce reelin in other postnatal structures.

We used different approaches to deplete the availability of reelin. We either blocked the secretion with BFA or the protein synthesis with cycloheximide. BFA treatment selectively abolished the fraction of punctate reelin IR whereas cycloheximide selectively decreased the intense reelin IR population. These data show that the intense reelin IR pattern is associated with synthesis and secreting functions. This interpretation is consistent with the observation that intense reelin IR neurons were not detected in non-permeabilized conditions suggesting that intense reelin IR is intracellular (not shown). One might expect treatment with cycloheximide to diminish the punctate reelin IR population by limiting the pool of synthesized reelin available for binding. Several explanations can account for the lack of effect within the time scale of the experiment; the turnover rate of unbound reelin could be very slow or alternatively, the pool of secretable reelin may not be sufficiently depleted and and thus still be readily releasable even after synthesis have been blocked for a long period of time. Finally, our observation is compatible with reelin recycling in punctate reelin IR neurons since it is known that after binding to lipoprotein receptors, reelin undergoes endocytosis [2], [38]. BFA can inhibit recycling whereas cycloheximide should not affect this mechanism, consistent with our observation that BFA but not cycloheximide decreased the fraction of punctate reelin IR neurons.

The present data and our previous work [18], show that ApoER2 is expressed in most hippocampal neurons and that VLDLR-positive neurons represent a much smaller population (25%). As a consequence, a large fraction of hippocampal neurons would be expected to display a punctate IR pattern. In contrast, at the plateau of reelin expression, the neuronal population associated to receptor-bound reelin represented a small percentage of hippocampal neurons (22%). This could be due to the presence of neurons that do not reach the detection threshold for immunocytochemistry or that levels of unbound reelin in the culture medium is not high enough to bind all receptors. Strickingly, the fractions of punctate reelin IR neurons and VLDLR-positive neurons are very similar, and reelin could preferentially bind to neurons expressing VLDLR or both VLDLR and ApoER2. In support of this hypothesis, we observed that all punctate reelin IR neurons express VLDLR.

Several studies have shown that, in the postnatal and adult brain, reelin modulates neuronal plasticity, including long-term potentiation, dendritic branching, maturation and surface diffusion of NMDARs [12], [15]–[18]. However, whether the continuous secretion of reelin is required for its physiological function at postnatal stages was never addressed. So far, BFA is the only inhibitor known to block reelin secretion [11], thus we treated hippocampal cultures with BFA to assay the physiological consequences of the lack of reelin secretion. Blockade of the secretory pathway perturbed the homeostasis of NMDARs and changed the NR2B-/NR2A-NMDARs ratio. BFA could either block the basal insertion of NR2A-NMDARs or increase the number of functional NR2B-NMDARs, therefore enhancing in both cases the fraction of ifenprodil sensitive-NMDARs. Noteworthy, this effect was reversed following the addition of endogenous or recombinant reelin showing that the continuous secretion of reelin is necessary to maintain the subunit composition of NMDA receptors.

Several molecular mechanisms could account for the effects of reelin secretion. First, the activation of the reelin signaling pathway could result in the phosphorylation of NMDARs thus upregulating NR2B-dependent responses [23]. The fact that a long incubation period with endogenous or recombinant reelin is needed to rescue secretion blockade does not favor this hypothesis. Second, we have previously shown that reelin controls the surface mobility of both synaptic and extrasynaptic NR2B-containing NMDARs [12]. We reported that the lack of reelin, selectively affected the surface mobility of NR2B- versus NR2A-containing NMDARs. Reelin depletion significantly decreased the surface mobility of extrasynaptic NR2B-containing NMDARs [12]. The effect of increasing the availability of NR2B-containing NMDARs could in turn contribute to the enhancement the fraction of functional NR2B-containing receptors. Finally, the fact that reelin is a protein of the extracellular matrix suggests that its site of action may be located in perineuronal nets. Blockade of the secretory pathway perturbed the homeostasis of NMDARs and selectively abolished one type of reelin IR. These data not only suggest that diffusion of reelin is crucial for its physiological action, but also favor the hypothesis that reelin diffuses broadly into the interstitial space to act as a trans-neuronal messenger. This does not rule out a possible autocrine function for reelin through cell surface diffusion and/or vesicular transport.

Based on our data, we propose that defects in reelin secretion could play a major role in the development of neuropsychiatric disorders. Particularly those associated with deregulation of NMDARs homeostasis and lower expression levels of reelin, such as schizophrenia [39], [40].

## Materials and Methods

### Hippocampal cultures

Hippocampi were dissected from postnatal day 0 Swiss mice brains. First, tissues were enzymatically dissociated for 40 min at 37°C in HBSS containing papain (20 units/ml, Sigma-Aldrich). Papain was then inactivated with Trypsin Inhibitor (125 mM, Sigma-Aldrich) in the presence of bovine serum albumin (0.25%, Sigma-Aldrich). Hippocampi were then mechanically triturated in the culture medium. Finally, dissociated cells were plated on poly-L-lysine coated round coverslips (10 µg/ml, Sigma-Aldrich) at a density of 462 per mm^2^. Neurons were grown in Minimum Essential Medium (MEM, GIBCO Invitrogen) supplemented with 5% of Serum Supreme (Biowhittaker), 28.3 mM D-glucose and 0.1% of Mito+ Serum Extender (Becton Dickinson). Media were fully renewed the day after plating and exchanged weekly. Cultures were kept in a humidified atmosphere of 5%CO_2_-95%O_2_ at 36.5°C. To avoid glial cell multiplication, cultures were treated at 9 day in vitro (div) with 0.2 mg/ml 5′-fluoro-2-deoxyuridine and 0.5 mg/ml uridine (FUDR, Sigma-Aldrich).

### Animals

All experiments were conducted in strict compliance with European directives and French laws on animal experimentation (authorization number 3307016).

### Treatment of cultures

Brefeldin A (BFA, Sigma-Aldrich) was used as previously described (0.75 µg/ml) [41]. Absolute ethanol was used as a control and added to the same final volume percentage. For immunocytochemistry, BFA treatments were performed between 12 and 14 div. After 9 h, coverslips were washed 3 times with serum-free culture medium and returned to the incubator during 10 hours for recovery before fixation. For calcium imaging, neurons were treated with BFA for 15 h and imaged at 9, 10 and 15 div. For recovery experiments, BFA was washed after 9 to 15 h incubation and neurons were imaged 24 h to 28 h later at 11 and 15 div. Rescue experiments were performed after a 9 h treatment with BFA, by adding conditioned medium collected from 16–17 div cultures or recombinant reelin, in the presence of BFA for the following 13 hours. Neurons were then recorded at 9, 10 and 11 div. Recombinant reelin and the mock control were produced as described previously [12]. The presence of reelin in conditioned medium and in the recombinant fraction were assessed by western blot.

Cultures were treated at 9 div for 24 hours with different concentrations - 25, 40 and 50 µg/ml - of either the Receptor-Associated Protein fused with glutathione S-transferase (GST-RAP) or the control GST [18].

Cycloheximide (Sigma-Aldrich) treatments were performed at 13 div. Cycloheximide was added directly to the culture medium at a final concentration of 10 µM. Absolute ethanol with the same final volume percentage was used as a control.

### Immunocytochemistry

Hippocampal cultures were fixed in 4% paraformaldehyde-containing Phosphate Buffer Solution (PBS), pH 7.4 for 40 min at room temperature. Quenching was performed with 0.1 M glycine (Sigma-Aldrich) in PBS for 45 min. Permeabilization was achieved with PBS containing 0.1% triton X-100 (Sigma-Aldrich) for 5 min or PBS containing 0.05% saponin for 30 min. Non specific binding was blocked with 2 sequential incubations of 15 min duration with blocking buffer. Blocking buffer consisted of PBS containing either 10% donkey serum (Chemicon) or 0.2% Bovin Serum Albumin (Sigma-Aldrich) plus 0.05% saponin. Bovin Serum Albumin was used at 1% with anti-ApoER2 and anti-VLDLR primary antibodies. Cells were incubated overnight at 4°C in a humidified atmosphere with the primary antibodies diluted in blocking buffer. Preparations were then washed twice 15 min followed by a 30 min rinse in blocking buffer and incubated with the secondary antibodies for 80 min at room temperature in a humidified atmosphere. Cells were then rinsed extensively with the blocking buffer and mounted on glass coverslips in Aqua-PolyMount (PolySciences).

Reelin was detected with the monoclonal antibody G10 (1∶2000, Chemicon) and visualized with a cyanine 3 (Cy3)-conjugated anti-mouse IgG (1∶500, Chemicon) or an Alexa Fluor 568-conjugated anti-mouse IgG (1∶500, Molecular Probes). MAP2 was revealed with a rabbit polyclonal antibody (1∶500) and visualized with Alexa Fluor 647-conjugated anti-rabbit IgG (1∶500, Molecular Probes). To visualize GABAergic neurons, we used antibodies directed against different isoforms of the GABA synthesizing enzymes, the rabbit anti-Glutamic Acid Decarboxylase 67 (GAD67, 1∶1000, Chemicon) and the rabbit anti-GAD65 (1∶1000, Chemicon). These primary antibodies were revealed with cyanine 5 (Cy5)-conjugated anti-rabbit IgG (1∶500, Jackson Immunoresearch) or Alexa Fluor 488-conjugated anti-rabbit IgG (1∶500, Molecular Probes). The vesicular glutamate transporters 1 and 2 were detected with rabbit anti-VGlut1 (1∶1000) or VGlut2 (1∶2500) respectively and revealed with Cy5-conjugated anti-rabbit IgG (1∶500, Jackson Immunoresearch). Reelin lipoprotein receptors were labeled with the rabbit anti-ApoER2 (1∶200, Santa Cruz Biotechnology) and the mouse anti-VLDLR (1∶500, Santa Cruz Biotechnology) antibodies revealed with Alexa Fluor 488 anti-rabbit IgG (1∶500, Molecular Probes) and Cy3-conjugated anti-mouse IgG (1∶500, Jackson Immunoresearch) respectively.

### Image analysis of immunostaining

Slides were viewed with a 40× oil objective on an Axiophot II microscope (Zeiss, France). Image acquisition was done with a Quantix digital cooled CCD camera (Photometrix, Tucson, AZ) driven by IPLab software (Scanalytics, Fairfax, VA, USA). The exposure settings were kept identical for each condition. For each fluorescent image that was acquired, the corresponding phase-contrast field was collected. The 12 bit images were analysed with MetaMorph software 7.1.2.0 (Universal Imaging, Evry, France) as follows. Somata were demarcated on the phase-contrast image, and the regions of interest (ROIs) were transferred automatically on the corresponding fluorescent images. Positive ROIs were selected using intensity thresholds determined by the distribution of intensities obtained amongst different experiments. Positive somata were then counted over all of the images acquired on coverslips from one experiment and normalized to the total number of neurons detected on the corresponding phase-contrast images.

### Calcium imaging and analysis

Hippocampal neurons were loaded with 2 µM Oregon Green 488 BAPTA-1 AM (Molecular Probes) for 30 min at 37°C in culture medium. Coverslips were then washed 4 times with the Mg^2+^-free recording medium containing (in mM): NaCl (160), KCl (2.4), Hepes (10), glucose (10), glycine (0.02) and CaCl2 (3), pH = 7.3 and Osm = 325 mOsm/l. Coverslips were then mounted onto a custom made recording/perfusion chamber positioned on the movable stage of an inverted microscope Olympus IX-71. Cells were superfused (0.7 ml/min) with the recording medium. Signals were detected using a CCD camera (Quantem 512SC) and acquired with the Metamorph software 7.1.2.0 (Universal Imaging Corp). Images were acquired with the filter set 41001, HQ 480/40 (Chroma Technology, Brattelboro, VT, USA) for excitation and HQ 535/50 (Chroma Technology) for emission. Recording sessions were between 40 and 45 minutes. The acquisition rate was 0,017 Hz during baseline (10–12 min) and ifenprodil application (3 µM; 3 min) and 0.5 Hz during NMDA (100 µM) and NMDA (100 µM)+ifenprodil (3 µM) applications. Phase contrast images were acquired prior and after each experiment to control for cellular integrity.

Data were analysed using the Metamorph software 7.1.2.0. After background correction, positive ROIs were demarcated only on somata whose integrity was confirmed in phase contrast images. Raw values (Ft) were normalized to the average of baseline calculated before NMDA application (Fr) and data were plotted as ΔF/F = (Ft−Fr)/Fr. NMDA responses were calculated as ΔF/F (NMDA±ifen)−ΔF/F (before NMDA±ifen).

### Statistical analysis

All experiments were performed at least in 3 different cultures, n is the number of cultures used for each condition unless stated otherwise. All data are expressed as the mean±SEM. Statistical analysis were performed by ANOVA followed by Tukey's multiple comparison test where significant (P<0.05, Kyplot 2.0β1.3).

## Supporting Information

Figure S1 Time course of reelin immunostaining during treatment with BFA or vehicle. (A) Representative images of intense and punctate reelin IR after 3 hours and 16 hours of BFA treatment and after BFA recovery. (B) Treatment with vehicle does not affect the percentage of reelin immunoreactive neurons at various time points. Values for intense reelin IR were: before treatment (0 hr) 21.1±1.2%, n = 8; 3 hours 19.7±2.0%, n = 3; 6 hours 21.2±1.0%, n = 3; 9 hours 18.8±1.3%, n = 5; 16 hours 21.2±0.4%, n = 6 and recovery 21.3±2.6%, n = 6. Values for punctate reelin IR were: untreated (0 hr) 18.0±1.4%, n = 8; 3 hours 17.7±2.5%, n = 3; 6 hours 19.0±1.6%, n = 3; 9 hours 18.1±1.4%, n = 5; 16 hours 17.4±1.1%, n = 6 and recovery 17.7±0.2%, n = 6.
(6.53 MB TIF)

Figure S2 Secretion blockade decreases the levels of total and full-length reelin present in the medium of cultured hippocampal neurons. (A) Example western blot of the time course of BFA treatment on reelin secreted in the medium of 13 div hippocampal cell cultures. Bands corresponding to full-length reelin (400 kDa) and proteolytic products (320 and 180 kDa) were disclosed by the G10 antibody. Reelin was not detected in medium not exposed to cultured neurons (medium). (B, C) Time course of the effect of BFA treatment on the levels of secreted reelin measured by western blot. For each condition, values are expressed as the percentage±sem of the corresponding vehicle control. **P<0.01, ***P<0.001. (B) Densitometry measurements of total reelin expressed as the sum of the densities of the 3 forms, full-length reelin (400 kDa) plus the two reelin fragments (320 and 180 kDa). A significant decrease is observed after 3 hours (53.8±2.9% of vehicle) and 9 hours (41.3±0.4% of vehicle) of treatment compared to 1 hour treatment with BFA (93.9±2.1% of vehicle) or vehicle (n = 3; F(3,8) = 7.7, P<0.01). (C) Densitometry measurements of the 400 kDa band, corresponding to full-length reelin, after treatment with either vehicle, 1 hour BFA (84.8±3.0%), 3 hours BFA (42.8±4.0%) or 9 hours BFA (22.7±0.4%; n = 3; F(3,8) = 99.1, P<0.001).
(5.90 MB TIF)

Figure S3 Time course of reelin immunostaining during treatment with cycloheximide or vehicle. (A) Representative images showing intense and punctate reelin IR after 3, 9 and 16 hours of cycloheximide treatment (CHX). (B) The percentage of reelin immunoreactive neurons was not changed during incubation with vehicle at various time points. Values for intense reelin IR were: before treatment 23.7±1.7%, n = 4; 1 hour 21.7±1.7%, n = 3; 3 hours 19.1±2.7%, n = 3; 6 hours 18.7±2.9%, n = 4; 9 hours 20.2±3.1%, n = 3 and 16 hours 19.2±1.4%, n = 3. (C) Values for punctate reelin IR were: before treatment 18.4±0.8%, n = 4; 1 hour 18.2±2.6%, n = 3; 3 hours 17.9±2.4%, n = 3; 6 hours 19.7±3.1%, n = 4; 9 hours 17.6±1.0%, n = 3 and 16 hours 17.1±6.2%, n = 3.
(8.10 MB TIF)

Figure S4 Dual labelling of reelin and VLDLR. Representative image of a punctate reelin IR neuron (A) showing co-expression of VLDLR (B) in a 14 div hippocampal culture.
(1.62 MB TIF)

Figure S5 Subsequent applications of NMDA do not induce run-down of Ca2+ responses. Example of Ca2+ responses kinetics recorded in a 10 div neuron during subsequent applications of NMDA. The intensity of Ca2+ response remained stable during consecutive NMDA applications. On average the amplitude of the Ca2+ response obtained during the second application of NMDA represented 94.5±3.3% of the response evoked by the first application of NMDA (n = 33 neurons).
(1.56 MB TIF)

Table S1 Supplementary Table
(0.03 MB DOC)

Text S1 Supporting Materials
(0.04 MB DOC)

## References

[1] D'Arcangelo G, Nakajima K, Miyata T, Ogawa M, Mikoshiba K (1997). Reelin is a secreted glycoprotein recognized by the CR-50 monoclonal antibody.. J Neurosci.

[2] D'Arcangelo G, Homayouni R, Keshvara L, Rice DS, Sheldon M (1999). Reelin is a ligand for lipoprotein receptors.. Neuron.

[3] Herz J, Chen Y (2006). Reelin, lipoprotein receptors and synaptic plasticity.. Nat Rev Neurosci.

[4] Dulabon L, Olson EC, Taglienti MG, Eisenhuth S, McGrath B (2000). Reelin binds alpha3beta1 integrin and inhibits neuronal migration.. Neuron.

[5] Alcantara S, Ruiz M, D'Arcangelo G, Ezan F, de Lecea L (1998). Regional and cellular patterns of reelin mRNA expression in the forebrain of the developing and adult mouse.. J Neurosci.

[6] Drakew A, Frotscher M, Deller T, Ogawa M, Heimrich B (1998). Developmental distribution of a reeler gene-related antigen in the rat hippocampal formation visualized by CR-50 immunocytochemistry.. Neuroscience.

[7] Derer P, Derer M (1990). Cajal-Retzius cell ontogenesis and death in mouse brain visualized with horseradish peroxidase and electron microscopy.. Neuroscience.

[8] Ikeda Y, Terashima T (1997). Expression of reelin, the gene responsible for the reeler mutation, in embryonic development and adulthood in the mouse.. Dev Dyn.

[9] Ramos-Moreno T, Galazo MJ, Porrero C, Martinez-Cerdeno V, Clasca F (2006). Extracellular matrix molecules and synaptic plasticity: immunomapping of intracellular and secreted Reelin in the adult rat brain.. Eur J Neurosci.

[10] Pesold C, Impagnatiello F, Pisu MG, Uzunov DP, Costa E (1998). Reelin is preferentially expressed in neurons synthesizing gamma-aminobutyric acid in cortex and hippocampus of adult rats.. Proc Natl Acad Sci U S A.

[11] Lacor PN, Grayson DR, Auta J, Sugaya I, Costa E (2000). Reelin secretion from glutamatergic neurons in culture is independent from neurotransmitter regulation.. Proc Natl Acad Sci U S A.

[12] Groc L, Choquet D, Stephenson FA, Verrier D, Manzoni OJ (2007). NMDA receptor surface trafficking and synaptic subunit composition are developmentally regulated by the extracellular matrix protein Reelin.. J Neurosci.

[13] Ogawa M, Miyata T, Nakajima K, Yagyu K, Seike M (1995). The reeler gene-associated antigen on Cajal-Retzius neurons is a crucial molecule for laminar organization of cortical neurons.. Neuron.

[14] Tissir F, Goffinet AM (2003). Reelin and brain development.. Nat Rev Neurosci.

[15] Beffert U, Weeber EJ, Durudas A, Qiu S, Masiulis I (2005). Modulation of synaptic plasticity and memory by Reelin involves differential splicing of the lipoprotein receptor Apoer2.. Neuron.

[16] Weeber EJ, Beffert U, Jones C, Christian JM, Forster E (2002). Reelin and ApoE receptors cooperate to enhance hippocampal synaptic plasticity and learning.. J Biol Chem.

[17] Niu S, Renfro A, Quattrocchi CC, Sheldon M, D'Arcangelo G (2004). Reelin promotes hippocampal dendrite development through the VLDLR/ApoER2-Dab1 pathway.. Neuron.

[18] Sinagra M, Verrier D, Frankova D, Korwek KM, Blahos J (2005). Reelin, very-low-density lipoprotein receptor, and apolipoprotein E receptor 2 control somatic NMDA receptor composition during hippocampal maturation in vitro.. J Neurosci.

[19] Lippincott-Schwartz J, Donaldson JG, Schweizer A, Berger EG, Hauri HP (1990). Microtubule-dependent retrograde transport of proteins into the ER in the presence of brefeldin A suggests an ER recycling pathway.. Cell.

[20] De A, Krueger JM, Simasko SM (2003). Tumor necrosis factor alpha increases cytosolic calcium responses to AMPA and KCl in primary cultures of rat hippocampal neurons.. Brain Res.

[21] Herz J, Goldstein JL, Strickland DK, Ho YK, Brown MS (1991). 39-kDa protein modulates binding of ligands to low density lipoprotein receptor-related protein/alpha 2-macroglobulin receptor.. J Biol Chem.

[22] Hiesberger T, Trommsdorff M, Howell BW, Goffinet A, Mumby MC (1999). Direct binding of Reelin to VLDL receptor and ApoE receptor 2 induces tyrosine phosphorylation of disabled-1 and modulates tau phosphorylation.. Neuron.

[23] Qiu S, Zhao LF, Korwek KM, Weeber EJ (2006). Differential reelin-induced enhancement of NMDA and AMPA receptor activity in the adult hippocampus.. J Neurosci.

[24] Chavis P, Westbrook G (2001). Integrins mediate functional pre- and postsynaptic maturation at a hippocampal synapse.. Nature.

[25] Cull-Candy SG, Leszkiewicz DN (2004). Role of distinct NMDA receptor subtypes at central synapses.. Sci STKE.

[26] Hestrin S (1992). Developmental regulation of NMDA receptor-mediated synaptic currents at a central synapse.. Nature.

[27] Monyer H, Burnashev N, Laurie DJ, Sakmann B, Seeburg PH (1994). Developmental and regional expression in the rat brain and functional properties of four NMDA receptors.. Neuron.

[28] Stocca G, Vicini S (1998). Increased contribution of NR2A subunit to synaptic NMDA receptors in developing rat cortical neurons.. J Physiol.

[29] Bellone C, Nicoll RA (2007). Rapid bidirectional switching of synaptic NMDA receptors.. Neuron.

[30] Carmignoto G, Vicini S (1992). Activity-dependent decrease in NMDA receptor responses during development of the visual cortex.. Science.

[31] Philpot BD, Sekhar AK, Shouval HZ, Bear MF (2001). Visual experience and deprivation bidirectionally modify the composition and function of NMDA receptors in visual cortex.. Neuron.

[32] Williams K (1993). Ifenprodil discriminates subtypes of the N-methyl-D-aspartate receptor: selectivity and mechanisms at recombinant heteromeric receptors.. Mol Pharm.

[33] Sheng M, Cummings J, Roldan LA, Jan YN, Jan LY (1994). Changing subunit composition of heteromeric NMDA receptors during development of rat cortex.. Nature.

[34] Tovar KR, Westbrook GL (1999). The incorporation of NMDA receptors with a distinct subunit composition at nascent hippocampal synapses in vitro.. J Neurosci.

[35] Martinez-Cerdeno V, Galazo MJ, Cavada C, Clasca F (2002). Reelin immunoreactivity in the adult primate brain: intracellular localization in projecting and local circuit neurons of the cerebral cortex, hippocampus and subcortical regions.. Cereb Cortex.

[36] Hevner RF, Neogi T, Englund C, Daza RA, Fink A (2003). Cajal-Retzius cells in the mouse: transcription factors, neurotransmitters, and birthdays suggest a pallial origin.. Brain Res Dev Brain Res.

[37] Ina A, Sugiyama M, Konno J, Yoshida S, Ohmomo H (2007). Cajal-Retzius cells and subplate neurons differentially express vesicular glutamate transporters 1 and 2 during development of mouse cortex.. Eur J Neurosci.

[38] Jossin Y, Gui L, Goffinet AM (2007). Processing of Reelin by embryonic neurons is important for function in tissue but not in dissociated cultured neurons.. J Neurosci.

[39] Guidotti A, Auta J, Davis JM, Di-Giorgi-Gerevini V, Dwivedi Y (2000). Decrease in reelin and glutamic acid decarboxylase67 (GAD67) expression in schizophrenia and bipolar disorder: a postmortem brain study.. Arch Gen Psychiatry.

[40] Mohn AR, Gainetdinov RR, Caron MG, Koller BH (1999). Mice with reduced NMDA receptor expression display behaviors related to schizophrenia.. Cell.

[41] Wisco D, Anderson ED, Chang MC, Norden C, Boiko T (2003). Uncovering multiple axonal targeting pathways in hippocampal neurons.. J Cell Biol.

