# Supplementary figures and images for: Reelin Secreted by GABAergic Neurons Regulates Glutamate Receptor Homeostasis
Source: PLoS One. 2009 May 11;4(5):e5505. doi: 10.1371/journal.pone.0005505 (PMC2675077; doi:10.1371/journal.pone.0005505)

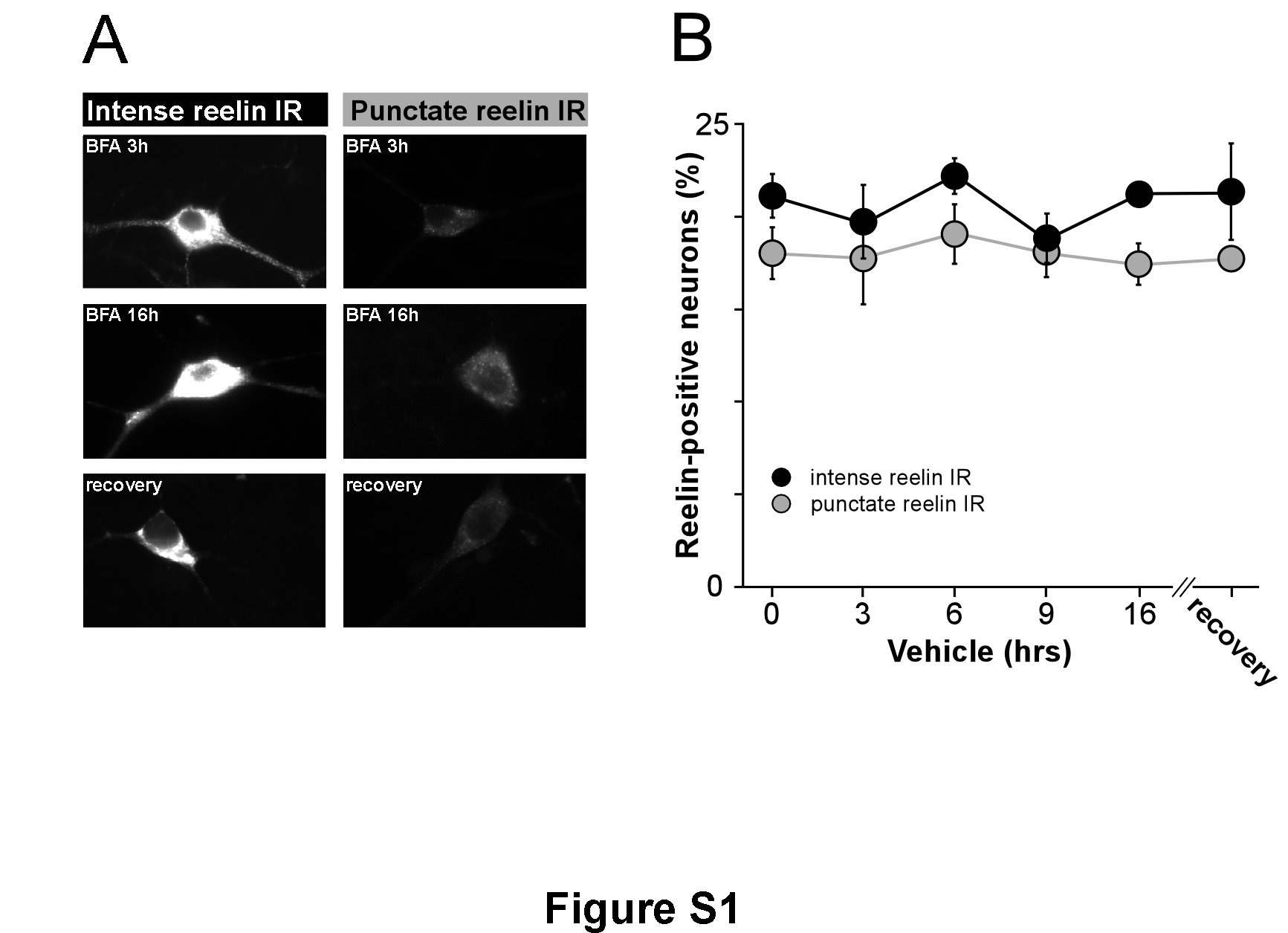

Supplement: Figure S1 — Time course of reelin immunostaining during treatment with BFA or vehicle. (A) Representative images of intense and punctate reelin IR after 3 hours and 16 hours of BFA treatment and after BFA recovery. (B) Treatment with vehicle does not affect the percentage of reelin immunoreactive neurons at various time points. Values for intense reelin IR were: before treatment (0 hr) 21.1±1.2%, n = 8; 3 hours 19.7±2.0%, n = 3; 6 hours 21.2±1.0%, n = 3; 9 hours 18.8±1.3%, n = 5; 16 hours 21.2±0.4%, n = 6 and recovery 21.3±2.6%, n = 6. Values for punctate reelin IR were: untreated (0 hr) 18.0±1.4%, n = 8; 3 hours 17.7±2.5%, n = 3; 6 hours 19.0±1.6%, n = 3; 9 hours 18.1±1.4%, n = 5; 16 hours 17.4±1.1%, n = 6 and recovery 17.7±0.2%, n = 6. (6.53 MB TIF) [file pone.0005505.s001.tif]

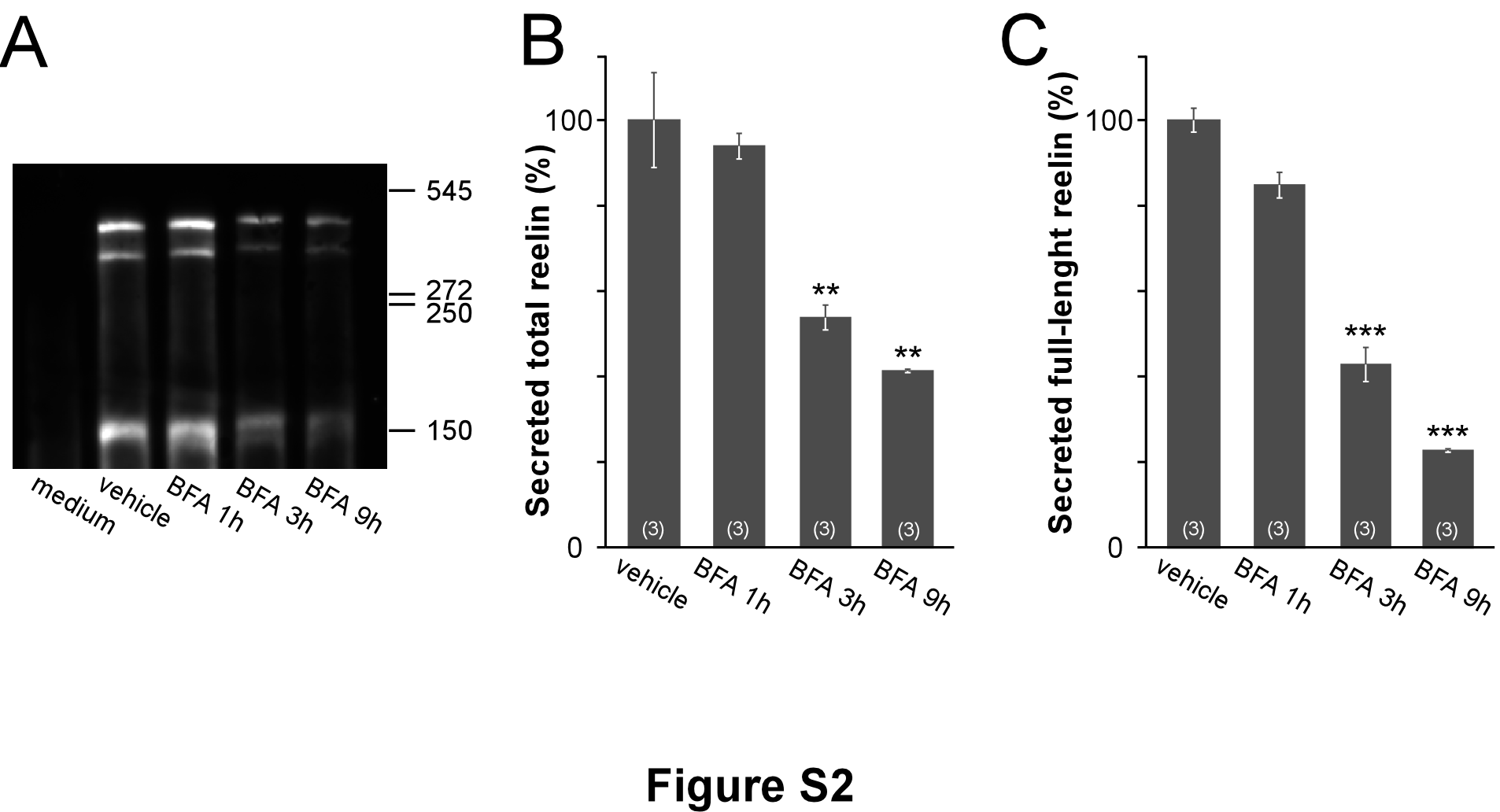

Supplement: Figure S2 — Secretion blockade decreases the levels of total and full-length reelin present in the medium of cultured hippocampal neurons. (A) Example western blot of the time course of BFA treatment on reelin secreted in the medium of 13 div hippocampal cell cultures. Bands corresponding to full-length reelin (400 kDa) and proteolytic products (320 and 180 kDa) were disclosed by the G10 antibody. Reelin was not detected in medium not exposed to cultured neurons (medium). (B, C) Time course of the effect of BFA treatment on the levels of secreted reelin measured by western blot. For each condition, values are expressed as the percentage±sem of the corresponding vehicle control. **P<0.01, ***P<0.001. (B) Densitometry measurements of total reelin expressed as the sum of the densities of the 3 forms, full-length reelin (400 kDa) plus the two reelin fragments (320 and 180 kDa). A significant decrease is observed after 3 hours (53.8±2.9% of vehicle) and 9 hours (41.3±0.4% of vehicle) of treatment compared to 1 hour treatment with BFA (93.9±2.1% of vehicle) or vehicle (n = 3; F(3,8) = 7.7, P<0.01). (C) Densitometry measurements of the 400 kDa band, corresponding to full-length reelin, after treatment with either vehicle, 1 hour BFA (84.8±3.0%), 3 hours BFA (42.8±4.0%) or 9 hours BFA (22.7±0.4%; n = 3; F(3,8) = 99.1, P<0.001). (5.90 MB TIF) [file pone.0005505.s002.tif]

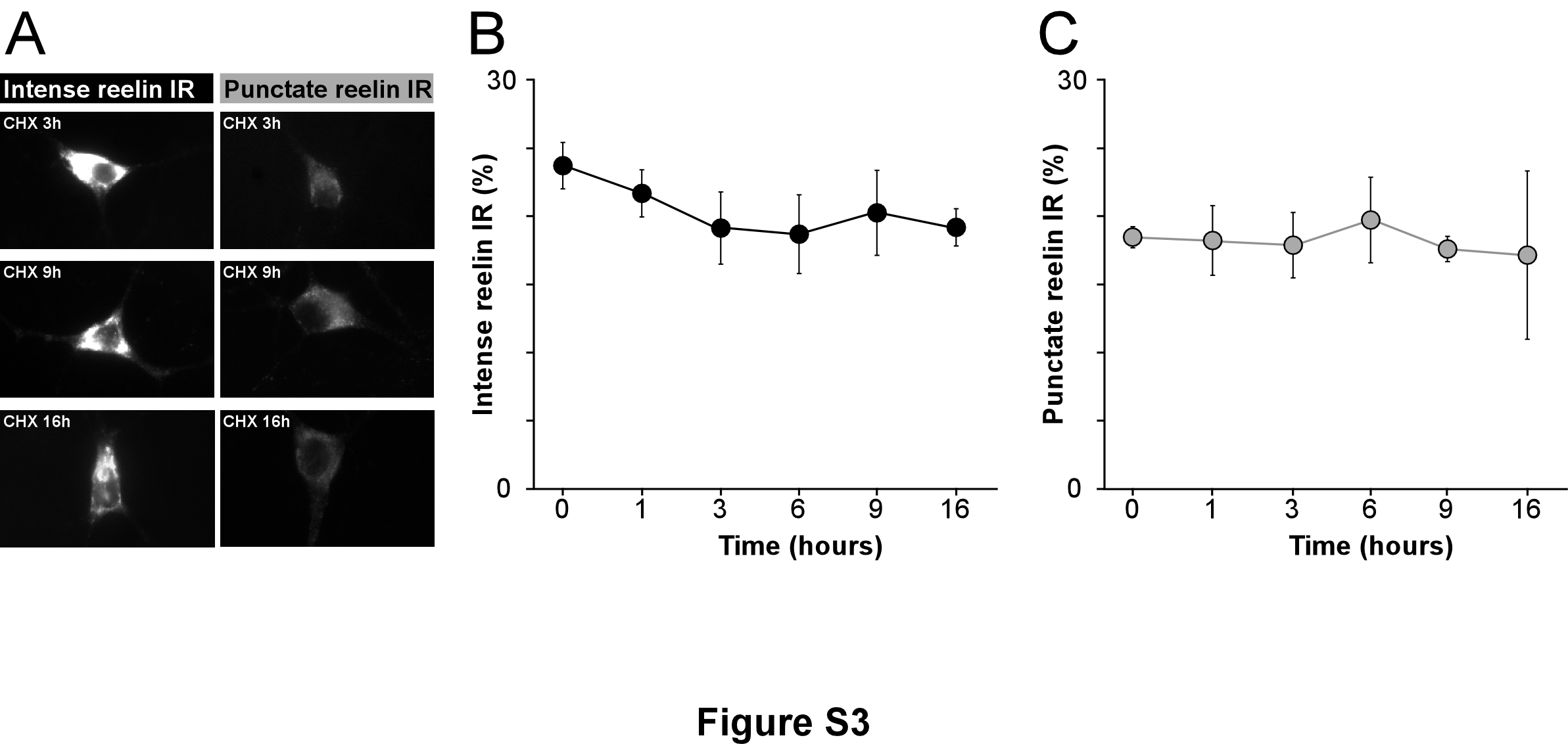

Supplement: Figure S3 — Time course of reelin immunostaining during treatment with cycloheximide or vehicle. (A) Representative images showing intense and punctate reelin IR after 3, 9 and 16 hours of cycloheximide treatment (CHX). (B) The percentage of reelin immunoreactive neurons was not changed during incubation with vehicle at various time points. Values for intense reelin IR were: before treatment 23.7±1.7%, n = 4; 1 hour 21.7±1.7%, n = 3; 3 hours 19.1±2.7%, n = 3; 6 hours 18.7±2.9%, n = 4; 9 hours 20.2±3.1%, n = 3 and 16 hours 19.2±1.4%, n = 3. (C) Values for punctate reelin IR were: before treatment 18.4±0.8%, n = 4; 1 hour 18.2±2.6%, n = 3; 3 hours 17.9±2.4%, n = 3; 6 hours 19.7±3.1%, n = 4; 9 hours 17.6±1.0%, n = 3 and 16 hours 17.1±6.2%, n = 3. (8.10 MB TIF) [file pone.0005505.s003.tif]

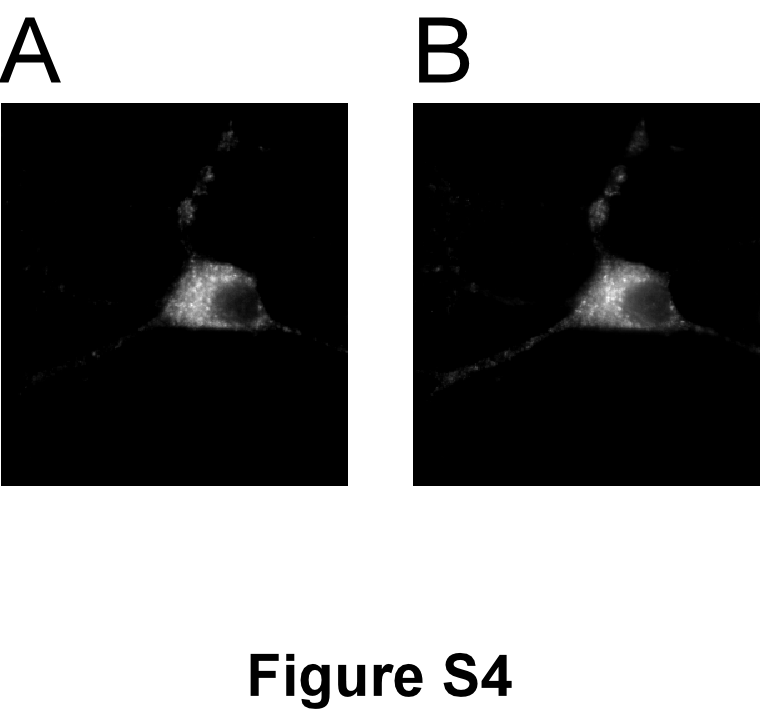

Supplement: Figure S4 — Dual labelling of reelin and VLDLR. Representative image of a punctate reelin IR neuron (A) showing co-expression of VLDLR (B) in a 14 div hippocampal culture. (1.62 MB TIF) [file pone.0005505.s004.tif]

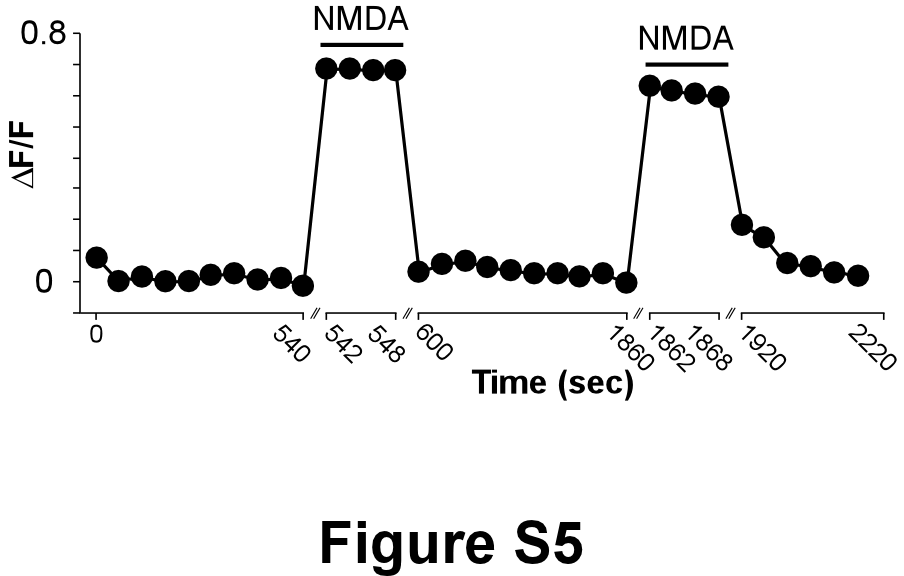

Supplement: Figure S5 — Subsequent applications of NMDA do not induce run-down of Ca2+ responses. Example of Ca2+ responses kinetics recorded in a 10 div neuron during subsequent applications of NMDA. The intensity of Ca2+ response remained stable during consecutive NMDA applications. On average the amplitude of the Ca2+ response obtained during the second application of NMDA represented 94.5±3.3% of the response evoked by the first application of NMDA (n = 33 neurons). (1.56 MB TIF) [file pone.0005505.s005.tif]
