# Supplementary material for: Reelin Secreted by GABAergic Neurons Regulates Glutamate Receptor Homeostasis
Source: PLoS One. 2009 May 11;4(5):e5505. doi: 10.1371/journal.pone.0005505 (PMC2675077; doi:10.1371/journal.pone.0005505)
Supplement: Table S1 — Supplementary Table (0.03 MB DOC) [file pone.0005505.s006.doc]

**SUPPLEMENTARY TABLE**

**Table S1. Blockade of integrins does not affect the proportion of intense and punctate reelin IR neurons.**

| **Treatment** | **% intense reelin IR**  **± sem** | **% punctate reelin IR ± sem** | **n** |
| --- | --- | --- | --- |
| Untreated | 17.0 ± 0.1 | 14.6 ± 3.1 | 3 |
| GRGDSP | 15.6 ± 1.8 | 14.3 ± 0.9 | 3 |
| GRGESP | 16.8 ± 2.1 | 16.8 ± 2.1 | 3 |

9 div neurons were chronically treated either with the synthetic peptide GRGDSP (200µM) that disrupts the binding of ECM ligands to β1- or β3-containing integrins, or the control peptide GRGESP (200µM).
