# Supplementary material for: Reelin Secreted by GABAergic Neurons Regulates Glutamate Receptor Homeostasis
Source: PLoS One. 2009 May 11;4(5):e5505. doi: 10.1371/journal.pone.0005505 (PMC2675077; doi:10.1371/journal.pone.0005505)
Supplement: Text S1 — Supporting Materials (0.04 MB DOC) [file pone.0005505.s007.doc]

**SUPPLEMENTARY METHODS**

**Immunoblotting of secreted reelin.** Western blotting of reelin with the G10 antibody and processing of dishes were performed as described previously [11,17]. Samples of 50 µg of homogenate proteins run through the same 6% SDS-PAGE were subjected at the same time to protein quantification with the BCA protein assay kit (Pierce, Rockford). Treatments with BFA and vehicle were performed as described in materials and methods. Treatments and processing of culture dishes were performed following a double-blind protocol to avoid any bias. Immunoblots chemiluminescence was acquired with ChemiGenius 2 (SynGene, Ozyme) and band densities quantified with GeneTools (SynGene, Ozyme). In each lane, total reelin was calculated as the sum of the density measurements of the 400 kDa (full-length reelin), 320 kDa and 180 kDa bands. For each gel, the density measurements in BFA conditions for total and full-length reelin were normalized to the corresponding vehicle conditions.
